# Mesenchymal stem cells enhance the metastasis of 3D-cultured hepatocellular carcinoma cells

**DOI:** 10.1186/s12885-016-2595-4

**Published:** 2016-07-30

**Authors:** Chang Liu, Yang Liu, Xiao-xi Xu, Xin Guo, Guang-wei Sun, Xiao-jun Ma

**Affiliations:** 1 Laboratory of Biotechnology, Dalian Institute of Chemical Physics, Chinese Academy of Sciences, 457 Zhongshan Road, Dalian, 116023 People’s Republic of China; 2 University of Chinese Academy of Sciences, Beijing, 100049 China

**Keywords:** Three-dimensional cell culture, Umbilical cord mensenchymal stem cells, Hepatocellular carcinoma, Metastasis, TGF-β

## Abstract

**Background:**

Accumulating evidences have demonstrated that mesenchymal stem cells (MSC) could be recruited to the tumor microenvironment. Umbilical cord mesenchymal stem cells (UCMSC) were attractive vehicles for delivering therapeutic agents against cancer. Nevertheless, the safety of UCMSC in the treatment of tumors including hepatocellular carcinoma (HCC) was still undetermined.

**Methods:**

In this study, an in vitro co-culture system was established to evaluate the effect of UCMSC on the cell growth, cancer stem cell (CSC) characteristics, drug resistance, metastasis of 3D-cultured HCC cells, and the underlying mechanism was also investigated.

**Results:**

It was found that after co-cultured with UCMSC, the metastatic ability of 3D-cultured HCC cells was significantly enhanced as indicated by up-regulation of matrix metalloproteinase (MMP), epithelial-mesenchymal transition (EMT)-related genes, and migration ability. However, cell growth, drug resistance and CSC-related gene expression of HCC cells were not affected by UCMSC. Moreover, EMT was reversed, MMP-2 expression was down-regulated, and migration ability of HCC cell was significantly inhibited when TGF-β receptor inhibitor SB431542 was added into the co-culture system.

**Conclusions:**

Therefore, these data indicated that UCMSC could significantly enhance the tumor cell metastasis, which was due to the EMT of HCC cells induced by TGF-β.

**Electronic supplementary material:**

The online version of this article (doi:10.1186/s12885-016-2595-4) contains supplementary material, which is available to authorized users.

## Background

Mesenchymal stem cells (MSC) are typically characterized by their ability to differentiate into a variety of mesenchymal cells. In recent years, MSCs have aroused a lot of interests due to their ability to give rise to bone, cartilage, fat, and muscle cells, which could be extensively used in regenerative medicine [1]. MSC reside in many adult organs or tissues, such as bone marrow (BM), adipose, fetal liver, lung, and umbilical cord (UC). UCMSC were attractive seed cells due to the least invasive source and their characteristics similar to those of BMMSC [2]. In addition, they have unique properties compared with other stem cells, such as high proliferation rate and hypoimmunogenicity [3].

There was growing evidence that MSC could be recruited to the injured sites in many pathological conditions, such as inflammation, tissue repair and tumor [4–6]. The migrating ability to tumor makes them useful as anti-tumor gene or drug carriers. The recent suggestion that MSC can be recruited by tumors has triggered a series of studies that aimed at examining their potential role in cancer progression. However, the effect of MSC on the tumor progression can be pro- [7–9] as well as anti-tumorigenic [10, 11] due to the different source of MSC and the tumor models used [5].

Besides, the role UCMSC played in tumor progression was also controversial. A few studies suggested UCMSC could inhibit tumor growth [12–14]. Ayuzawa et al. found UCMSC attenuated breast cancer growth by attenuation of Erk-1/2 and PI3K/AKT signaling pathway [12]. Ohta et al. showed FST over-expressing human UCMSC significantly reduced the growth of breast cancer cells [13]. The results of Chao et al. showed that when co-cultured with UCMSC, breast cancer cell number decreased significantly, which was caused by the tumorigenesis suppressing ability of UCMSC. They found that UCMSC induced the apoptosis of breast cancer cells by direct cell contact or by cell-in-cell phenomenon after internalization [14]. Nevertheless, UCMSC have been also reported to promote esophageal carcinoma cancer growth and metastasis both in vivo and in vitro [15]. The results concerning the effect of UCMSC on tumor growth were still mixed, and most of the in vitro studies were carried out under two-dimensional (2D) culture conditions.

Currently, HCC was the third most deadly and fifth most common cancer worldwide [16]. A few studies showed that BMMSC could inhibit cell division of HCC cells and potentiate their death [17–19]. Still there were some studies found that BMMSC in the inflammatory microenvironment of HCC promoted the development of chemoresistance and metastasis of HCC cells [20, 21]. The paradoxical effect of BMMSC in HCC progression was currently poorly understood, as the in vitro investigation was mostly performed in 2D culture system. In those studies, HCC cells were directly co-cultured with MSC, or treated with conditioned medium of MSC as indirect co-culture, both of which failed to mimic the interaction between HCC cells and MSCs in HCC microenvironment in vivo. In addition, as promising vehicles for delivering therapeutic agents, the safety of UCMSC in HCC treatment remains to be determined.

In our previous study, we established a three-dimensional (3D) culture system with alginate gel (ALG) beads. In this 3D culture system, adhesion (intergrin β1, ICAM 1), and ECM-related (typeIand type IV collagen) gene expression in HCC cells were up-regulated compared with 2D culture and close to those in liver cancer tissue, which represented a in vivo-like HCC cell culture model [22].

So in this study, HCC cells were cultured in ALG beads, and then co-cultured with UCMSC. The aim of this study was to evaluate the effect of UCMSC on the growth, CSC characteristics, drug resistance and metastasis of human HCC cells and investigate the underlying mechanisms to figure out the role that UCMSC play in HCC progression.

## Methods

### Materials

All chemicals were purchased from Sigma-Aldrich (St. Louis, MO, USA) unless otherwise specified. SB-431542 was added to cells at a concentration of 20 μM. The sodium alginate (MW: 500 kDa, G/M ratio was 33:67) was purified by removing protein and endotoxin according to the protocol used in our laboratory.

### Cell culture and encapsulation

HCC cell line, HCCLM3, was kindly provided by the Liver Cancer Institute, Zhongshan Hospital, Fudan University. HCCLM3 cells were maintained in high glucose Dulbecco’s Modified Eagle’s Medium (high glucose DMEM, Invitrogen, San Diego, CA) supplemented with 10 % fetal bovine serum (FBS) (HyClone, Logan, UT). Cells were encapsulated within ALG beads and cultured according to the previous study [23]. Cells were harvested from ALG beads by treatment with 55 mM sodium citrate. Images were taken using an inverted phase contrast microscope (Eclipse, Nikon, Tokyo, Japan) every other day.

Human UCMSC cells and the culture medium were kindly provided by Zhongyuan Union Stem Cell Bioengineering Corporation (Tianjin, China). Primary UCMSC were isolated according to the standard operating procedure of UC blood bank. Medium was changed every 3 days and cell passage was performed when 90 % confluence was reached.

### Co-culture of UCMSC and 3D-cultured HCCLM3

After 15-day culture in ALG beads, 3D cultured HCCLM3 cells (8 × 10^4^/cm^2^) were co-cultured with UCMSC (1.6 × 10^4^/cm^2^) for 5 days with or without SB431542 (20 μM) in flasks.

### Cell proliferation

Cell counting kit-8 (CCK8) (Dojindo Laboratories, Kumamoto, Japan) assay was use to detect cell proliferation according to the manufacturer’s instructions. The absorbance was recorded using a microplate reader (Well Scan MK3, Labsystems Dragon, Finland).

### Live/dead staining

ALG beads with HCCLM3 cells were collected and incubated with live/dead staining working solution composed of 2 μM calcein AM and 4 μM ethidium homodimer-1 (ED-1) at 37 °C for 2 h. After washing with normal saline, HCCLM3 cells were observed using a confocal laser scanning microscopy (CLSM) (SP2, Leica, Heidelberger, Germany).

### Quantitative real time RT-PCR

Real-time PCR was carried out with the SYBR Premix Ex Taq™ (Perfect Real Time) (Takara) method. Total RNA was isolated using RNAiso Plus (TaKaRa, Shiga, Japan) according to the manufacturer’s instruction. Reverse transcription was performed with the PrimeScript™ RT reagent kit (TaKaRa). PCR amplification and fluorescence detection were performed using the Mx3000P Real-Time Cycler (Agilent Technologies, Santa Clara, CA, USA). Primers (listed in Additional file 1: Table S1) were designed by Takara Biotechnology (Dalian) Co., Ltd. (Dalian, China). Each sample was tested in triplicate, and β-actin was used as an internal control. The results obtained from three independent experiments were presented as the calculated comparative expression ratios of target sample to 2D cell by using C_T_ method (2^-∆∆C^_T_).

### Drug resistance test

HCCLM3 cells cultured under different conditions were treated with cisplatin (5 μg/ml) for 48 h. The viability of surviving cells was measured by CCK8 assay.

### Zymography

Enzymatic activity of MMP2 and MMP9 was tested by gelatin zymography [22]. After incubation for 24 h, conditioned medium was collected and equal amounts of protein from each sample was loaded. MMP2 and MMP9 were differentiated according to their molecular weight, 72 kDa and 92 kDa, respectively.

### In vitro invasion assay

The invasiveness of HCCLM3 cells cultured under different conditions was evaluated by Matrigel invasion assay according to the previous study [24]. The transwell chambers (8 μm pore size) (Corning, Tewksbury, MA, USA) were coated with matrigel (BD, San Jose, CA, USA) according to the manufacturer’s instructions. HCCLM3 cells (10^5^ cells per insert) were seeded on the top of matrigel with serum-free DMEM. The lower chamber was filled with DMEM/10 % FBS. After incubated at 37 °C for 48 h, HCCLM3 cells migrated through the membrane were stained with crystal violet and counted under microscopic observation. The data were shown as the means ± SD of three independent assays.

### Statistical analysis

All experiments were performed three times independently as individual experiments. Data were expressed as means ± SD. Student’s *t*-test was used to determine the statistical significance between two groups. One-way ANOVA was used to specify differences between groups when more than two experimental groups were evaluated. Differences were considered to be significant for *p* < 0.05.

## Results

### Effect of UCMSC on the proliferation of 3D-cultured HCCLM3 cells

**Fig. 1:**
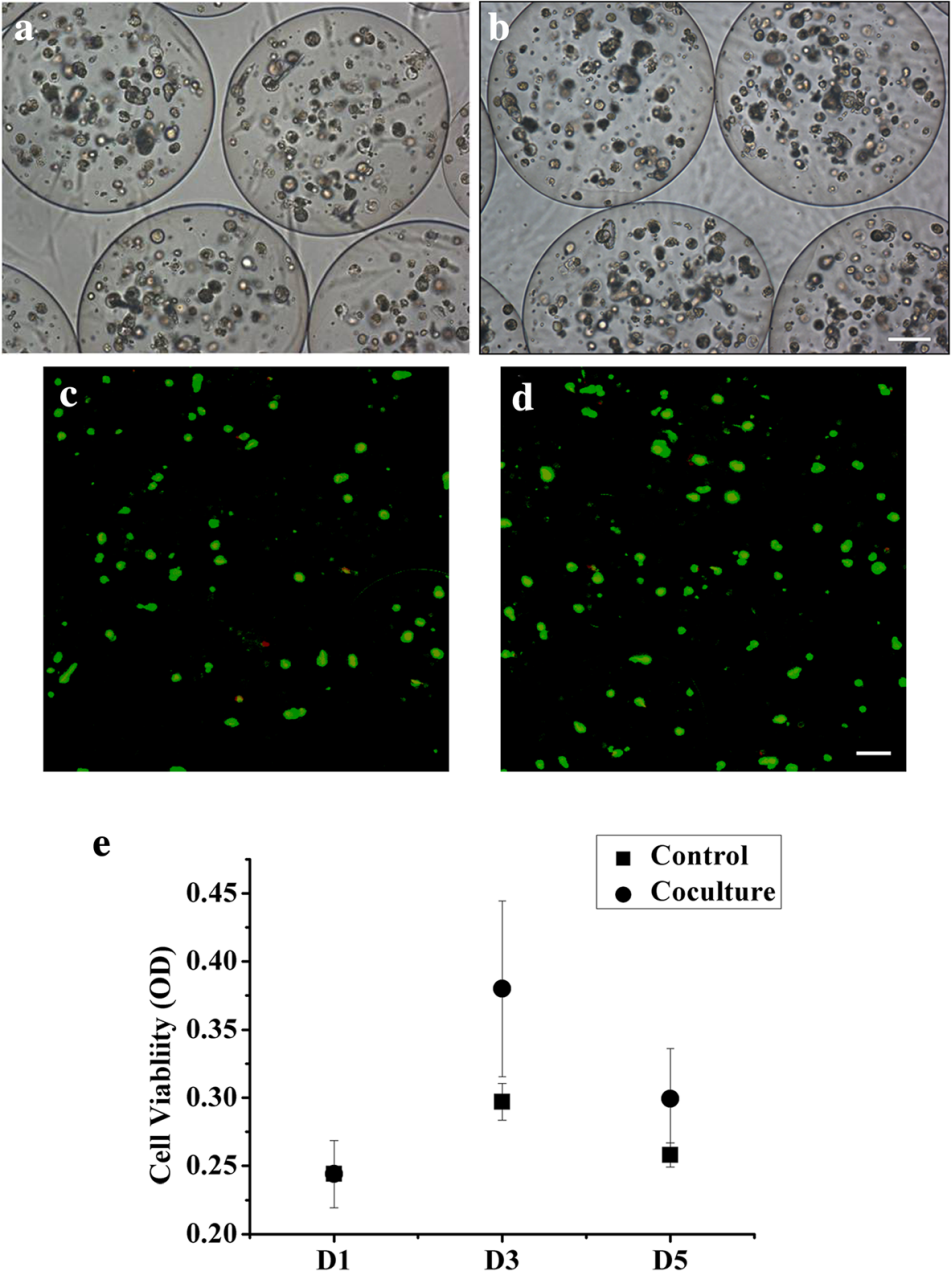
Morphology, viability and proliferation of 3D-cultured HCCLM3 cells. **a**-**b** Morphology and **c**-**d** live/dead staining of ALG beads-encapsulated HCCLM3 cells on Day 0 and Day 5 during co-culture. **e** Proliferation of 3D-cultured HCCLM3 cells in the co-culture and control group

Before co-cultured with UCMSC, the HCCLM3 cells were cultured in ALG beads for 15 days to form spheroids. The morphology (Day 0 and Day 5) during co-culture was shown in Fig. 1a-b. The results of live/dead staining suggested no obvious viability change during co-culture process (Fig. 1c-d). Cell proliferation assay showed a slight increase of cell viability during 5 days of co-culture, especially on Day 3, while no significant difference was found between these two groups (Fig. 1e). All of the above results indicated that the co-culture with UCMSC did not affect the cell viability and proliferation of 3D-cultured HCCLM3.

### Effect of UCMSC on CSC characteristics of 3D-cultured HCCLM3 cells

**Fig. 2:**
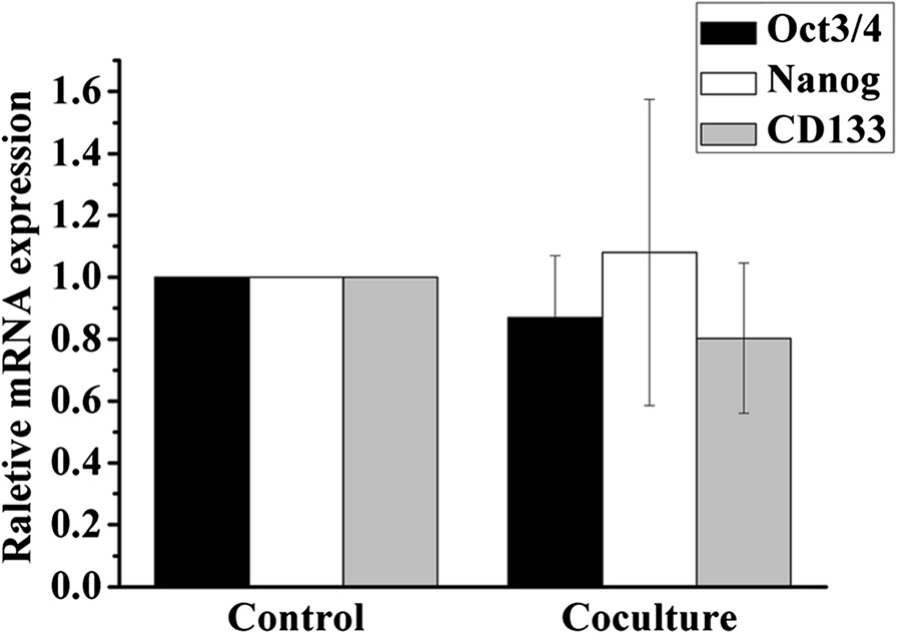
Expression of CSC-related genes of 3D-cultured HCCLM3 cells in the co-culture and control group

CSCs were reported to be the main culprit of cancer progression and metastasis [25]. Recently, it has been found that BMMSC can increase breast CSC population through cytokine loops in vivo [26]. To ascertain whether the co-culture with UCMSC had effect on CSC characteristics of HCC cells, the expression of stem cell marker in co-cultured HCC cells was investigated by real-time PCR analysis. No significant difference between co-culture group and control group in the expression of self-renew related genes, including Oct3/4 and Nanog, and HCC CSC surface marker CD133 (Fig. 2). These results indicated that UCMSC had no significant effect on the CSC enrichment of HCC cells.

### Effect of UCMSC on chemoresistance and metastatic properties of 3D-cultured HCCLM3 cells

**Fig. 3:**
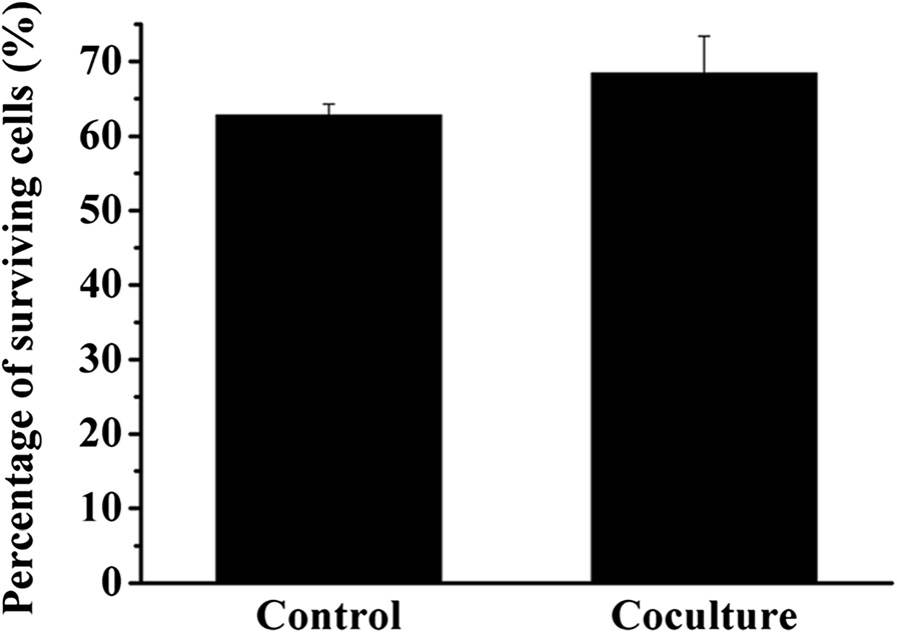
Percentage of surviving HCCLM3 cells after treated with cisplatin. Percentage of surviving 2D and 3D-cultured HCCLM3 cells in the co-culture and control group after treated with cisplatin. *p < 0.05, compared with 2D group

Development of resistance to chemotherapy and metastatic ability are major obstacles for lasting effective treatment of cancer. MSC in tumor microenvironment were reported to be closely related to drug resistance and metastasis of cancer [27–30]. In the following study, we assessed the chemoresistance and metastatic properties of HCCLM3 cells co-cultured with or without UCMSC. It showed that after treated with cisplatin, the percentage of surviving cells in control and co-culture group were significantly higher than 2D cultured cells (Fig. 3). In addition, although the percentage in co-culture group was higher than that in control group (68.43 % VS 62.85 %), no significant difference was found (Fig. 3).

**Fig. 4:**
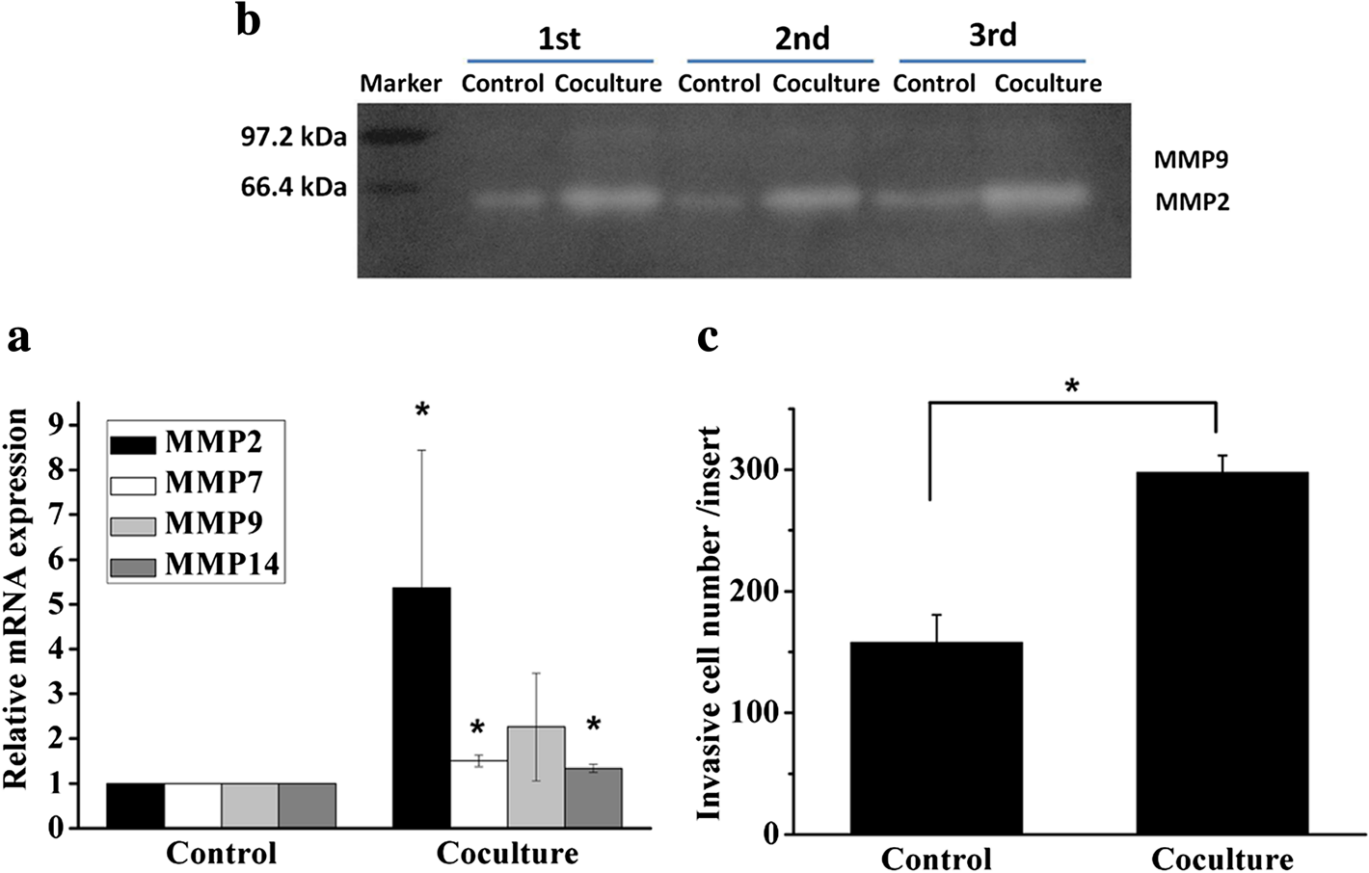
Gene and protein expression of MMPs in HCCLM3 cells. **a** Gene expression of MMP2, MMP7, MMP9 and MMP14 in the co-culture and control group. **b** Gelatin zymography detection of active MMP2 and MMP9 in three independent experiments. **c** Optical density analysis of active MMP2 expression. *p < 0.05, compared with the control group

**Fig. 5:**
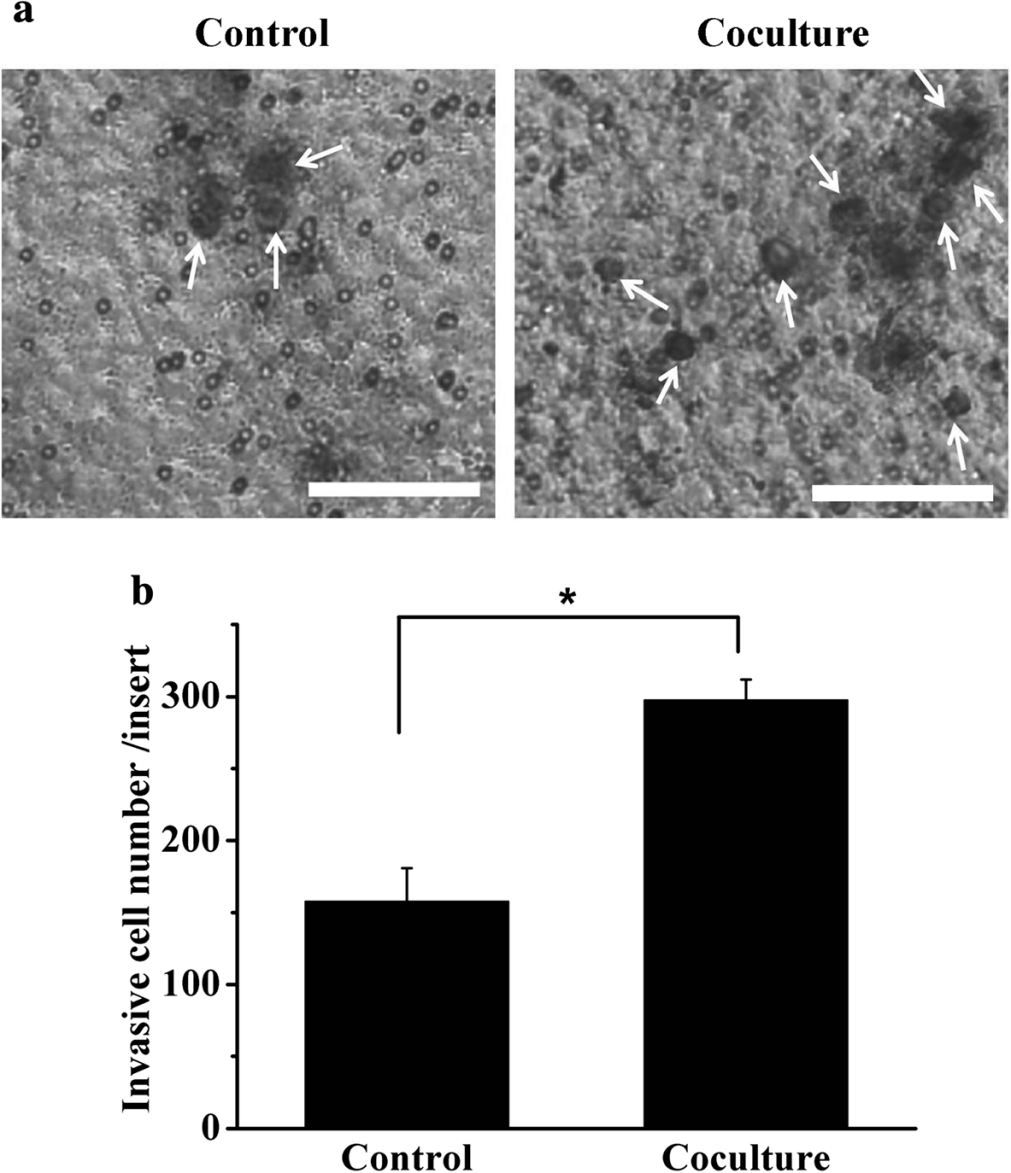
Matrigel invasion assay of HCCLM3 cells in the co-culture and control group. **a** HCCLM3 cells migrated through matrigel membrane, white arrows indicated cells stained with crystal violet. Bar:100 μm. **b** Total migrated HCCLM3 cells per transwell insert. *p < 0.05, compared with the control group

To better characterize the effect of UCMSC on the metastatic ability of 3D-cultured HCC cells, real-time PCR, zymography and in vitro invasion assay were performed. Significantly higher gene expression of MMP2, MMP7, and MMP14 were detected in co-cultured HCC cells compared with the control group (Fig. 4a). Furthermore, we also detected significantly higher expression of secreted active MMP2 protein in co-culture group compared with the control group (Fig. 4b). Although the relative mRNA expression of MMP9 in co-culture group was higher compared to the control, no significant different was detected (*P* = 0.062) (Fig. 4a). The active MMP9 in both co-culture and control group were at undetectable level (Fig. 4a), which was in accordance with our previous study [22]. Secretion of MMP2 promoted tumor cell invading through the matrigel layer which served as a reconstituted basement membrane in vitro [31]. Compared with the control group, the number of cells migrated through matrigel-coated membranes was increased by 1.89-fold in co-culture group (Fig. 5).

### Expression profile of EMT-related genes in co-cultured HCCLM3 cells

**Fig. 6:**
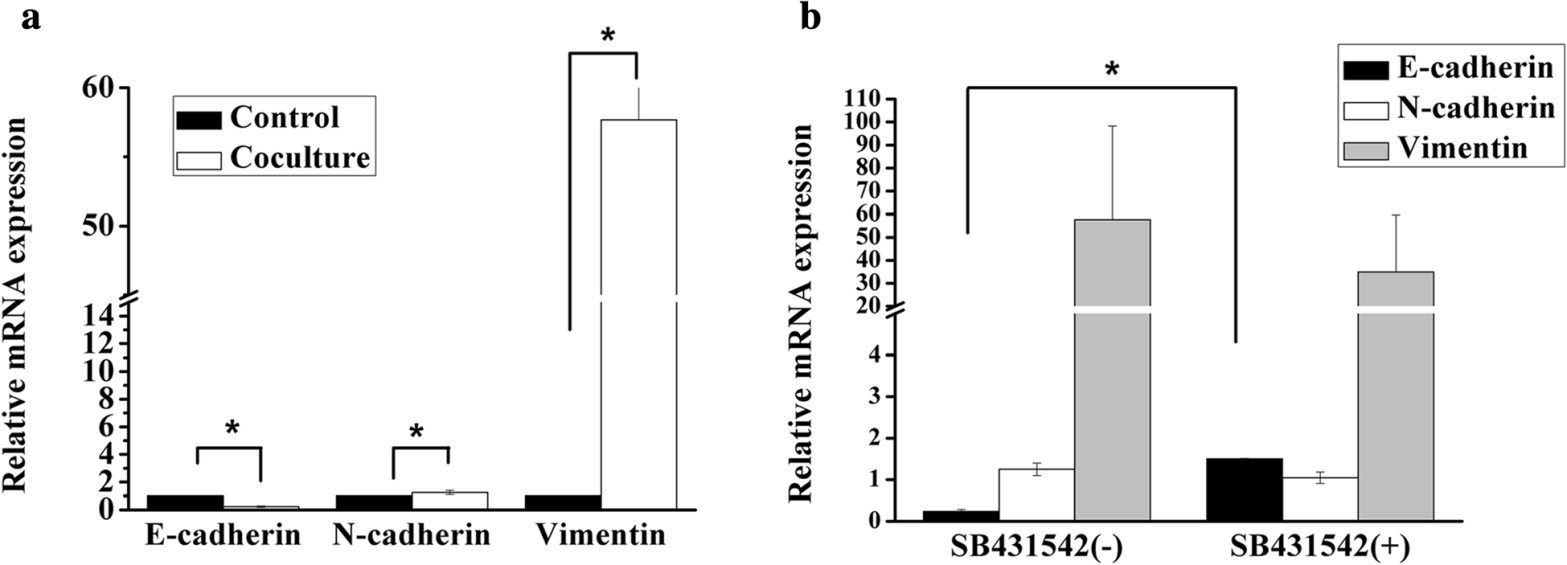
Expression profile of EMT-related genes in HCCLM3 cells. **a** EMT-related gene expression of HCCLM3 cells in the co-culture and control group. *p < 0.05, compared with the control group. **b** EMT-related genes of HCCLM3 cells after treated with TGF-β receptor antagonist SB431542. *p < 0.05, compared with the co-culture group without SB431542

During tumor progression, EMT contributed considerably to the malignant characteristics of tumors, such as local invasion and distant metastasis [32, 33]. In order to find out whether the enhancement of HCCLM3 metastasis was induced by EMT, we assessed the expression of EMT-related genes in different groups. The expression of N-cadherin and vimentin was significantly up-regulated in co-culture group with 1.25 ± 0.15-fold and 57.67 ± 40.63-fold separately, while E-cadherin was down-regulated with 0.23 ± 0.05-fold compared with that in the control group (Fig. 6a).

### Decreased metastatic ability of 3D-cultured HCCLM3 cells by inhibition of TGF-β in co-culture system

**Fig. 7:**
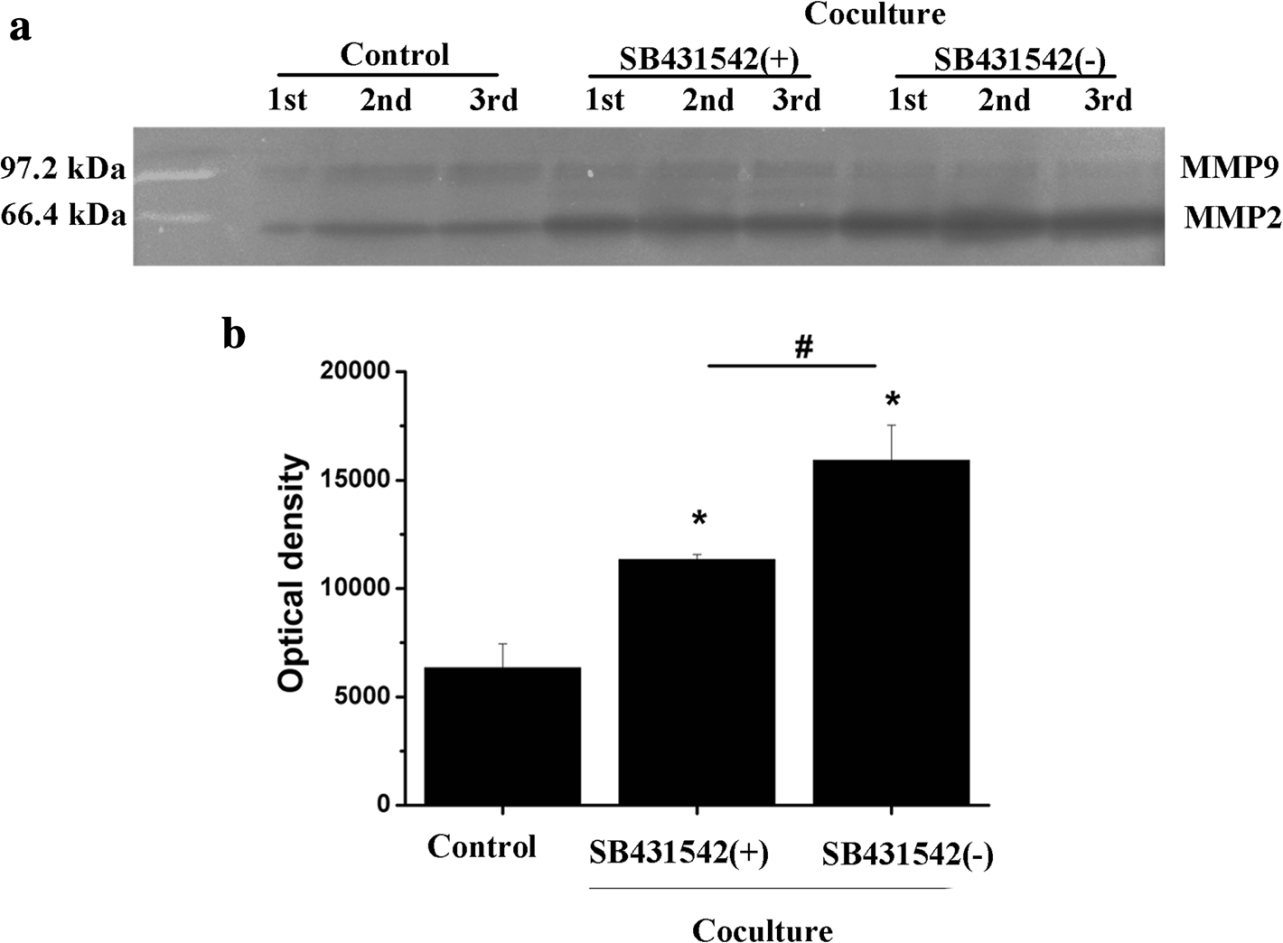
Active MMP2 expresison of HCCLM3 cells after treated with TGF-β receptor antagonist SB431542. **a** Gelatin zymography detection. **b** Optical density analysis of active MMP2 expression. *p < 0.05, compared with the control group. #*p* < 0.05, compared with the co-culture group

In this study, there was no direct interaction between 3D-cultured HCCLM3 cells and UCMSC, so the soluble factors in co-culture system might be responsible for the enhanced metastatic ability of HCC cells. It is confirmed that TGF-β can be secreted by both UCMSCs [34] and HCC cells [35]. In addition, several lines of evidence suggested increased TGF-β signaling as a key effector of EMT in HCC metastasis [36, 37]. Hence we tried to investigate the role that TGF-β played in the co-culture system. When TGF-β receptor antagonist SB431542 was added, increased E-cadherin, decreased N-cadherin and Vimentin expression were found compared with the control group (Fig. 6b), which suggested a reversed EMT process by inhibition of TGF-β in co-culture system. In addition, active MMP2 expression was inhibited (Fig. 7), and cell migration through matrigel-coated membrane was decreased (data not shown) when SB431542 was added. These results indicated that TGF-β was the key regulator of EMT and involved in the regulation of tumor cell metastasis by UCMSC.

## Discussion

For the less invasive source and lack of ethical concerns, UCMSC might be easily used as anti-tumor reagent delivery vehicle [38]. Nevertheless, the effect of UCMSC on tumor progression is still controversial, so the safety of UCMSC for cytotherapy was still undetermined. Almost all the in vitro studies on investigating the role of UCMSC in tumor development were performed under 2D cell culture conditions. As is well known, 3D-cultured tumor cells might more resemble the in vivo tumor tissue. So in this study, a co-culture system was used to evaluate the effect of UCMSC on the proliferation, CSC characteristics, drug resistance and metastatic ability of 3D-cultured HCCLM3 cells. And we found that HCC cell metastasis was significantly enhanced by UCMSC through TGF-β.

Jing et al. pretreated 2D-cultured BMMSC with IFNγ and TNFα to mimic the inflammation condition in tumor, and they found that the conditioned medium of MSC could promote the metastasis of HCC cell line SMMC-7721 and HeP-3B though the elevated expression of TGF-β, which induced the EMT of HCC cells [29]. In this study, our results supported their findings. However, Li et al. reported BMMSC inhibited the metastasis of HCC cell line MHCC97-H both in vitro and in vivo [11]. They found that the conditioned medium of 2D-cultured BMMSC enhanced in vitro proliferation, but suppressed the invasive ability of HCC cells through down-regulating TGF-β expression of HCC cells. These controversial findings might be attributed to the different cell lines and different co-culture methods. Cell lines originated from different stages of tumor development had distinct migration ability and might lead to the distinct reaction to the soluble factors in MSC conditioned medium. In addition, MSC cultured under normal and inflammation condition might secret different kinds of factors due to the different microenvironment where they resided. Therefore, further research should be performed to clarify the mechanism of BMMSC or UCMSC on tumor cell metastasis in different culture conditions.

ALG beads used in this work provided a 3D environment for HCCLM3 cells. After 15 days of culture, HCC cells formed tumor spheroids, which created a more in vivo-like tumor microenvironment. So we assumed that the secreted soluble factors of UCMSC would be more similar to that in vivo. Moreover, in this study, it was found that after co-cultured with UCMSC, TGF-β gene expression in HCCLM3 cells was almost the same as the control group (data not shown), which suggested that UCMSC didn’t affect the TGF-β expression of HCC cells. In addition, there was no direct contact between HCCLM3 cells and UCMSC in this study, so the secreted TGF-β in co-culture system would be the main culprit for the elevated metastatic ability of HCC cells.

Our results indicated that UCMSC would favor the metastasis of HCC cells, however, in vivo study should be performed to confirm it. Nevertheless, it should be cautious when using UCMSC as therapeutic vehicles, at least under hepatocellular carcinoma condition.

## Conclusions

In this study, a co-culture system was established to investigate the effect of UCMSC on 3D-cultured HCC cells. It was found that UCMSC could significantly enhance the metastasis of HCC cells by the induction of EMT which was regulated by TGF-β.

## Abbreviations

2D, two-dimensional; 3D, three-dimensional; ALG, alginate; BMMSC, bone marrow mesenchymal stem cell; CSC, cancer stem cell; EMT, epithelial to mesenchymal transition; HCC, hepatocellular carcinoma cell; MMP, matrix metalloproteinases; MSC, mesenchymal stem cell; UCMSC, umbilical cord mesenchymal stem cell

## Additional file

Additional file 1: Table S1. Primer pairs used for quantitative real-time PCR studies, including primer sequence, gene ID, and product length. (DOCX 14 kb)
