# Supplementary material for: Mesenchymal stem cells enhance the metastasis of 3D-cultured hepatocellular carcinoma cells
Source: BMC Cancer. 2016 Jul 30;16:566. doi: 10.1186/s12885-016-2595-4 (PMC4967520; doi:10.1186/s12885-016-2595-4)
Supplement: Additional file 1: Table S1. — Primer pairs used for quantitative real-time PCR studies, including primer sequence, gene ID, and product length. (DOCX 14 kb) [file 12885_2016_2595_MOESM1_ESM.docx]

**Table S.** Primer pairs used for quantitative real-time PCR studies.

| **Gene** | **Sequence(5’-3’)** | **Gene ID** | **Product length (bp)** |
| --- | --- | --- | --- |
| β-actin | TGGCACCCAGCACAATGAA  CTAAGTCATAGTCCGCCTAGAAGCA | NM_001101.3 | 187 |
| Oct3/4 | GACAGGGGGAGGGGAGGAGCTAGG  CTTCCCTCCAACCAGTTGCCCCAAAC | NM_002701.4 | 119 |
| Nanog | TCCAACATCCTGAACCTCAGCTA  AGTCGGGTTCACCAGGCATC | NM_024865.2 | 186 |
| CD133 | TGGATGCAGAACTTGACAACGT  ATACCTGCTACGACAGTCGTGGT | NM_001145852.1 | 133 |
| MMP2 | CTCATCGCAGATGCCTGGAA CAGCCTAGCCAGTCGGATTTG | NM_004530 | 167 |
| MMP9 | TGGGCTACGTGACCTATGACAT  GCCCAGCCCACCTCCACTCCTC | NM_004994.2 | 173 |
| MMP7 | GCATGAGTGAGCTACAGTGGGAAC  CCACATCTGGGCTTCTGCATTA | NM_002423.3 | 187 |
| MMP14 | GGAACCCTGTAGCTTTGTGTCTGTC  TGAGGGTCCTGCCTTCAAGTG | NM_004995.2 | 76 |
| E-cadherin | GAGTGCCAACTTGGACCATTCAGTA  AGTCACCCACCTCTAAGGCCATC | NM_004360.3 | 86 |
| N-cadherin | CGAATGGATGAAAGACCCATCC  GCCACTGCCTTCATAGTCAAACACT | NM_001792.3 | 171 |
| Vimentin | GGTGGACCAGCTAACCAACGA  TCAAGGTCAAGACGTGCCAGA | NM_003380.3 | 183 |
| TGF-β1 | GCGACTCGCCAGAGTGGTTA  GTTGATGTCCACTTGCAGTGTGTTA | NM_000660.4 | 143 |
|  |  |  |  |
